# Pig as a large animal model for posterior fossa surgery in oto-neurosurgery: A cadaveric study

**DOI:** 10.1371/journal.pone.0212855

**Published:** 2019-02-26

**Authors:** Mohamed Elsayed, Renato Torres, Olivier Sterkers, Daniele Bernardeschi, Yann Nguyen

**Affiliations:** 1 Sorbonne Université, Inserm, Unité "Réhabilitation chirurgicale mini-invasive et robotisée de l'audition", Paris, France; 2 AP-HP, GHU Pitié-Salpêtrière, Service ORL, Otologie, implants auditifs et chirurgie de la base du crâne, Paris, France; 3 Alexandria University, Faculty of Medicine, ORL Department, Alexandria, Egypt; Fraunhofer Research Institution of Marine Biotechnology, GERMANY

## Abstract

This study proposes a practical model for a new approach to the posterior fossa in common domestic pigs. Several surgical procedures can be simulated in the nonliving pig model, including soft tissue dissection, drilling of temporal bone, dural incision, access to the cerebellopontine angle, exposure of cranial nerves and drilling of the internal auditory canal. The pig model perfectly simulates standard otological and neurosurgical procedures, and we highlight the feasibility of our approach for further experiments in a living pig model with the possibility of reproducing the model for research on cranial nerves in pigs to study their electrophysiological behavior.

## Introduction

Many models have been described in the literature for otological and neurosurgical training and education including cadaveric dissection of human temporal bone, synthetic materials such as artificial temporal bones, animal models and recently, computer-based simulation models. The gold standard training is still human cadaveric temporal bone as this represents the most realistic model. Unfortunately, the availability of human temporal bone is becoming increasingly difficult and limited for legal, economic, and ethical reasons. Thus, temporal bones of animals, which are readily available at low cost, represent an excellent alternative [1, 2].

Additionally, living animal models are excellent candidates for investigation and electrophysiological evaluation of injury and regeneration processes of peripheral and cranial nerves. In our oto-neurosurgical daily practice, when we are dealing with pathologies located in the cerebellopontine angle (CPA) or at the skull base, surgical manipulations involving the facial and vestibulocochlear nerves carry a high risk of iatrogenic traumatic insult. This may result in severe functional loss of hearing and facial nerve palsy even though the nerves remain morphologically intact. Today, many animal models are well established for the investigation of peripheral nerve lesions; however, only a few studies have investigated cranial nerve injury [3].

Animal models involving small mammals such as rabbits, guinea pigs and rodents have been widely used. However, there is need for an animal model that better matches the human in size. Large animals such as the domestic pig and sheep are strong candidates. They can be used for practicing various procedures and also to improve our understanding of the basic anatomical structures and topography [4, 5].

Over the past few decades, several authors have studied and described the temporal bones and brain anatomy of pigs with the emergence of a pig experimental model that reflects the considerable resemblance to human anatomy and physiology [1, 4, 6–9]. Generally, pig brain is comparable to the human brain in gross anatomy. Homology exists in the relationship between pig and human skull base with a similar comparative anatomy with regard to brain, and upper and lower cranial nerves [10]. In addition, the weight of the adult pig brain ranges from 80 to 180 g which is more than 50 times greater than that of rat brain. Thus, the pig model can facilitate ideal and real life scenarios during neurosurgical training [7].

Therefore, studies have also described an in vivo pig model for general neurosurgical training. Surgical procedures have included craniotomy, dural opening, brain surgery and excision of an artificial tumor. Microscopy and bleeding management were also an integral part of training with the aim of developing a laboratory setting imitating an almost realistic operating room [11–13].

Despite this volume of available background data concerning pig temporal bones and pig brain anatomy, little attention has been paid to the possibility of using the pig as a surgical model in skull base surgery and specifically the posterior fossa. In 1999, Jarrahy et al. evaluated the anatomy of the CPA through a retro-sigmoid approach in a pig specimen but mainly to demonstrate its usefulness as a model for the use of endoscopy in skull base surgery [10].

Accordingly, our study aimed to assess the pig as a surgical model to access the posterior fossa with full exposure of the facial and vestibulocochlear nerves in the CPA and within the internal auditory canal (IAC) by establishing a detailed approach in pig specimens.

Our experiment highlights the feasibility of this approach for exposure of cranial nerves in the CPA and IAC in pigs which can subsequently be used as a possible in vivo model not only for oto-neurosurgical training but also for investigation and electrophysiological evaluation of cranial nerve injury.

## Methods

Seven adult heads of common domestic pigs (Sus scrofa domestica) were acquired from a regional slaughterhouse and dissected within 12 hours postmortem at our INSERM-UMR-S 1159 Laboratory, and two heads were dissected directly postmortem at the anatomy laboratory at Fer a Moulin Animal Hospital, Paris, France.

The aim of our approach was to expose the course of the facial and vestibulocochlear nerves emerging from the brain stem in the CPA and then within the IAC.

In all specimens, we performed the following steps: a skin incision, soft tissue dissection, craniotomy, dural opening in order to expose the CPA and visualize the acoustic-facial bundle, and finally drilling of the IAC to complete the exposure.

These steps were performed using an operating microscope (OpMi-1 Zeiss Inc., Jena, Germany), microsurgical instruments, suction, and a surgical drill.

## Results

### Purpose of the approach

Anatomically, the temporal bone in pigs is located in the same position as in humans; however, landmarks usually found in humans are missing. In our approach, the following structures could be identified: the posterior wall of the external auditory canal (EAC), the atlanto-occipital joint, the posterior arcade (which is synonymous with the posterior semicircular canal in humans [6]), the dura of the posterior cranial fossa, the posterior wall of the IAC and the acoustic-facial bundle.

The approach also allowed the drilling of the posterior wall of the EAC, the posterior arcade and the IAC, which are important surgical steps during surgery to remove tumors in the CPA.

Because of its relative simplicity, minimal invasiveness and satisfactory exposure of cranial nerves, this approach can be used in experimental animal studies specifically to access the CPA and IAC.

### Surgical procedure

**Fig 1:**
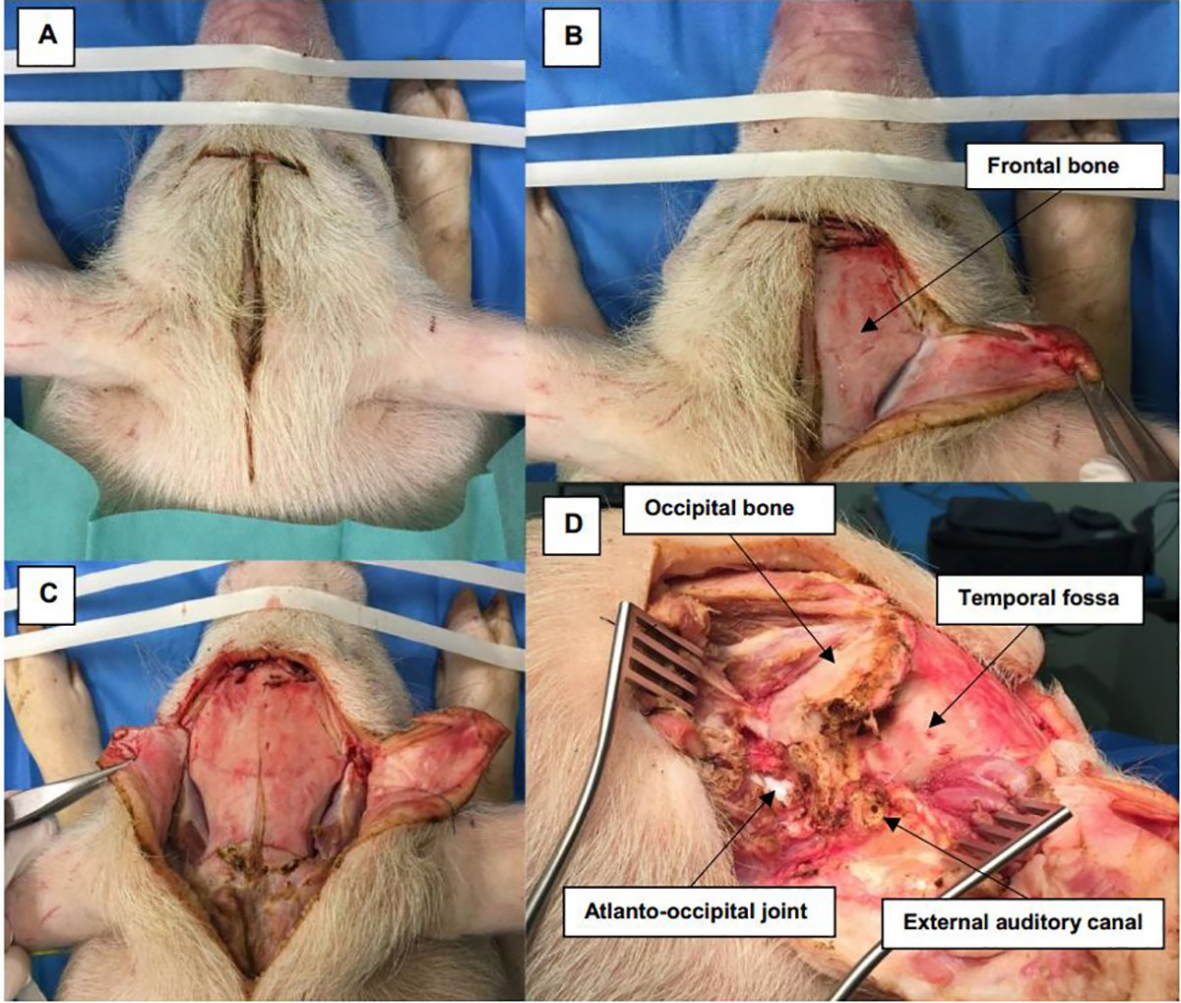
Incision and soft tissue dissection. (A) The pig’s head is in the prone position with a midline skin incision extending posteriorly then laterally into the post-auricular area. (B, C, D) Dissection of soft tissue over the frontal bone, temporal fossa (TF), occipital bone (OB), external auditory canal (EAC) and mastoid area, which is partially covered by the atlanto-occipital joint (AOJ).

**a. Incision and soft tissue dissection.** The pig’s head was positioned in the prone position. A midline vertical incision of skin and subcutaneous tissue was made between the eyes and extending posteriorly at the sloping crest of the occipital bone then laterally in a semilunar fashion through the post-auricular groove (Fig 1). Dissection of the skin flap continued laterally until the cartilaginous part of the EAC had been cut through.

Then, dissection of muscles overlying the occipital bone, temporal fossa and mastoid area in a sub-periosteal plane could be achieved easily. Separating muscle attachment on the occipital bone was done superiorly, laterally and at the midline. At the temporal fossa, we separated temporal muscle from its superior, posterior and inferior attachments until we had sufficiently exposed the parietal bone. We noted that, at the mastoid plane, this bone was partially covered by the atlanto-occipital joint [9]. Thus, it was necessary to dissect the muscle overlying it to expose part of the joint in order to facilitate subsequent drilling.

**b. Craniotomy.** Using different cutting and diamond burs, we started drilling following the direction of the posterior wall of the EAC as a landmark. The EAC is very long, narrow and oriented strictly upward and backward [9].

An important landmark was the posterior arcade, which could be easily identified following drilling of the EAC. It is the first arcade identified, not the lateral one as in humans. It is well and easily identified by the typical yellow color similar to the human otic capsule [6].

**Fig 2:**
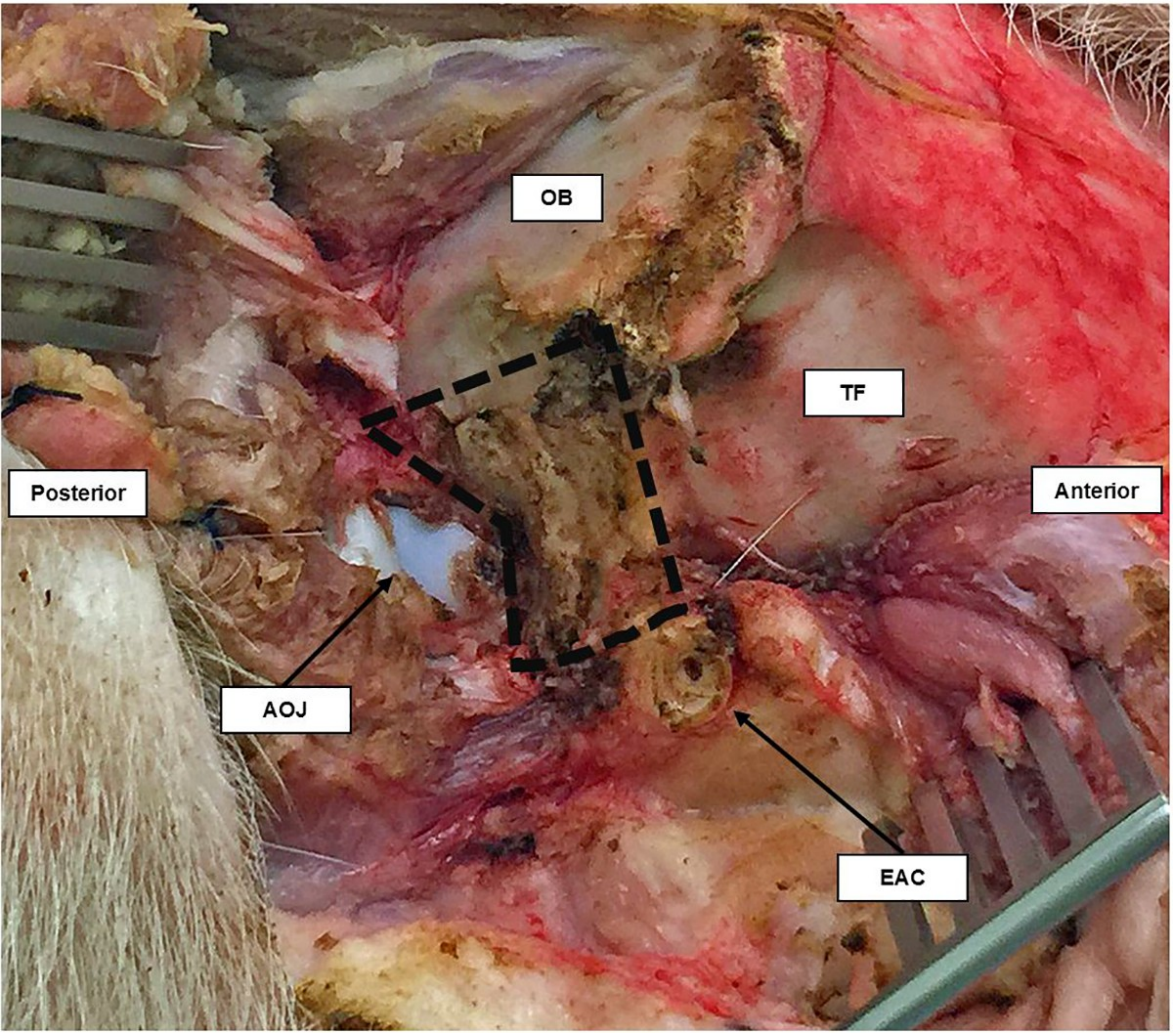
Lateral view of the right temporal bone of the pig. The area inside the black dashed line is the part of temporal bone covering the posterior fossa. It lies between the external auditory canal (EAC), occipital bone (OB) and atlanto-occipital joint (AOJ).

**Fig 3:**
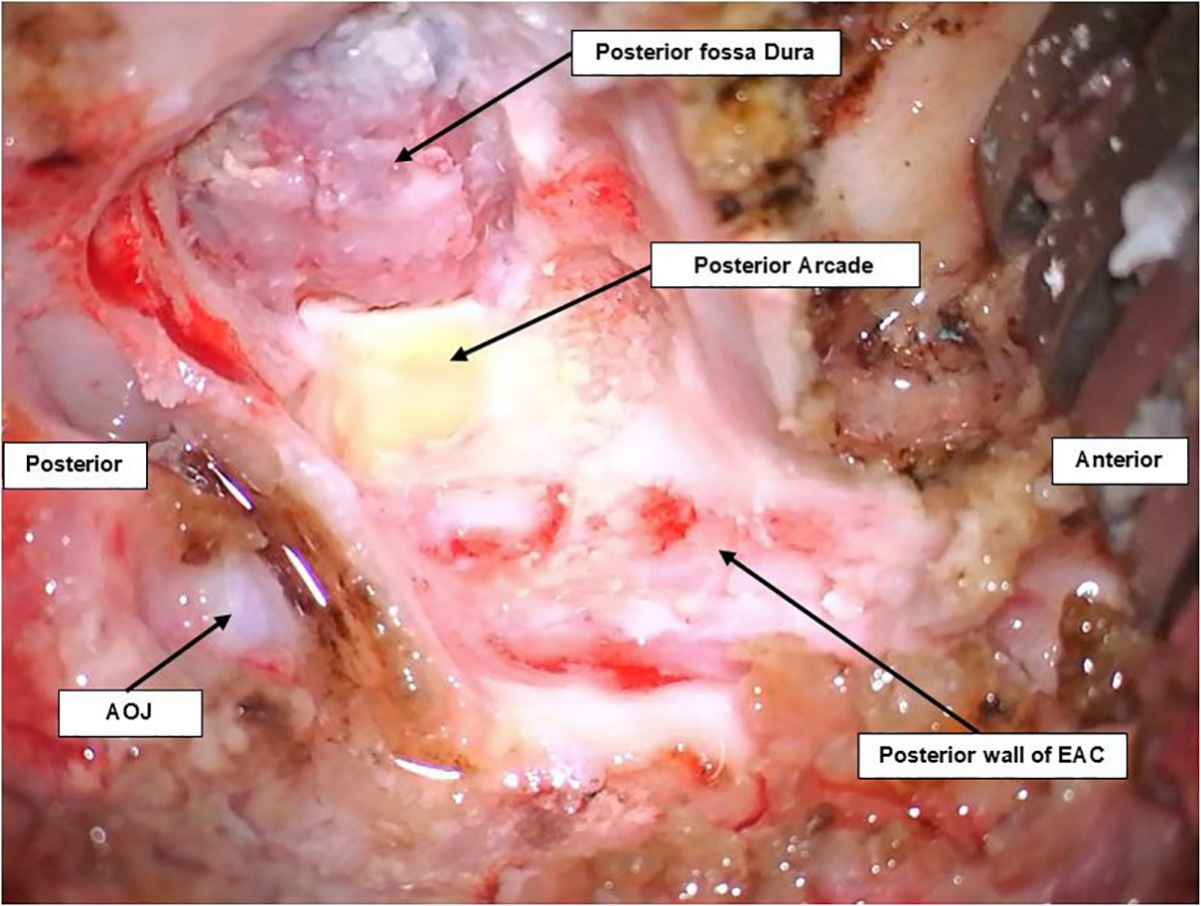
Lateral view of craniotomy of a pig’s ear on the right side. The posterior arcade can be seen as a yellow bone beside the dura of the posterior fossa. Both can be seen after drilling following the posterior wall of the EAC.

Medially, in a close anatomical relationship with the posterior arcade, the sigmoid sinus and the dura of the posterior fossa are in direct contact. Skeletonization of the dura was performed next using a diamond burr medially to the occipital bone and inferiorly to the atlanto-occipital joint. The mastoid air cells cannot be found in the pig because they are poorly pneumatized [5, 6], and are hidden under the atlanto-occipital joint (Figs 2 and 3).

**Fig 4:**
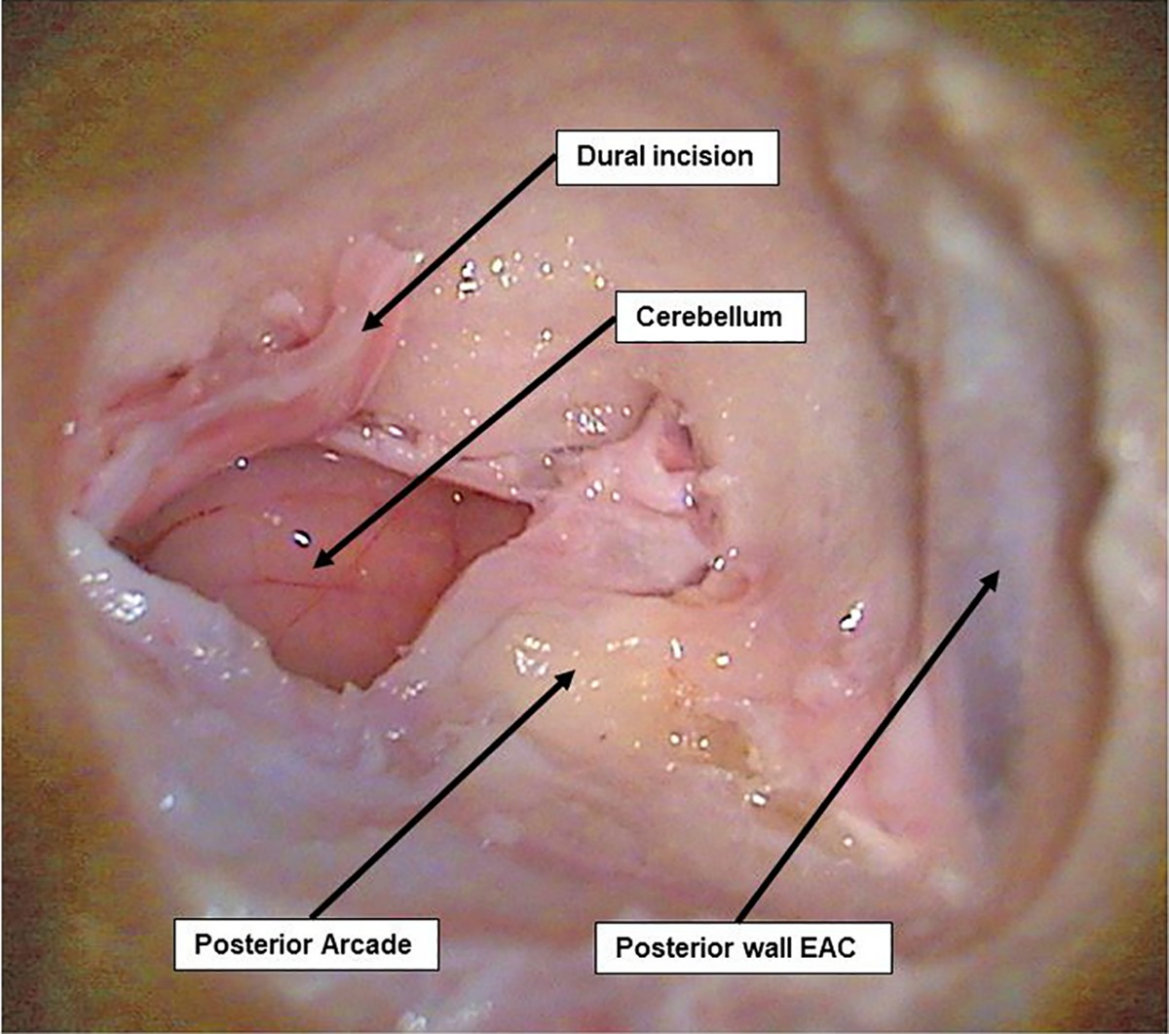
Dural incision and CPA exposure. Dural incision of the posterior fossa where the cerebellum can be easily identified and retracted.

**c. Access to CPA and IAC exposure.** After skeletonizing the dura over the posterior fossa, a dural incision was carefully performed with a posterior-based flap and brain tissue was exposed. The cerebellum was immediately identified and retracted posteriorly (Fig 4).

The acoustic-facial bundle (Cranial nerves VII/VIII) could be identified easily emerging from the pons and entering the IAC. As in humans, nerve bundles can be separated and the facial nerve lies anteriorly to the vestibulocochlear nerve.

**Fig 5:**
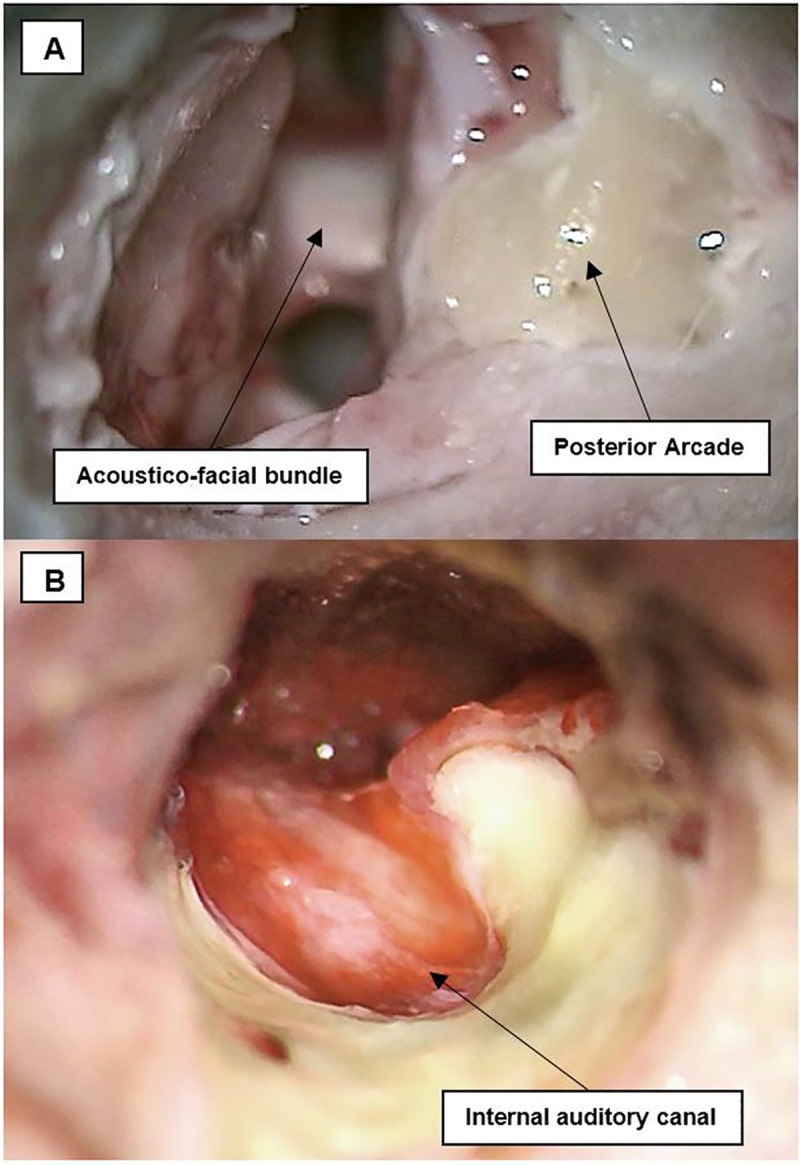
Acoustic-facial bundle in CPA and IAC exposure. (A) The acoustic-facial bundle (CN VII/VIII) could be seen emerging from the medulla, and entering the internal auditory canal (IAC). (B) After drilling of the posterior arcade and the posterior wall of the IAC, CN VII/VIII could be completely exposed.

**Fig 6:**
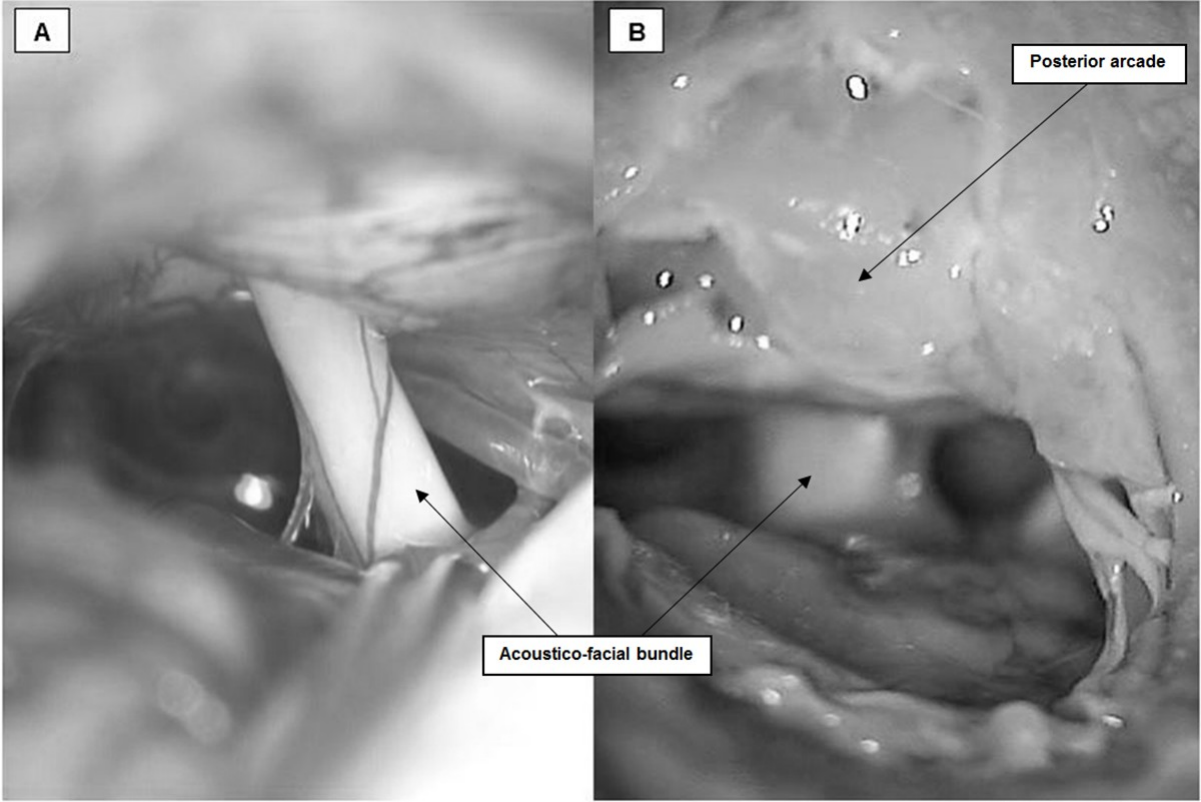
**Comparison between CPA microscopic view and acoustic-facial bundle in (A) human (courtesy of Dr Y. Nguyen) and (B) pigs**.

Further exposure of the facial and vestibulocochlear nerve bundle in the IAC could be achieved by first, careful drilling of the posterior arcade in order to allow more exposure and a better angle of view of the posterior wall of the IAC. Then, drilling of the IAC was performed slowly and deliberately using a diamond bur drill at low speed from a medial to lateral direction (Figs 5 and 6).

## Discussion

In the current literature, many studies have tried to find alternatives to human temporal bone in otological and neurosurgical training and research. Several models have been described, including nonliving animal models [1, 2, 6, 13–15]. These are readily available at low cost and may also offer a very realistic similarity to human anatomy in comparison to other synthetic materials. Temporal bones of sheep and pigs have been proposed by many authors as alternatives for otological education because the middle ear and tympanic membrane of both animals are morphologically similar to the structures found in human ears [2, 5].

Neurosurgical training models using pigs, sheep and cows have also been reported [1, 7, 10–12, 14–16]. Both nonliving and living models have been used extensively mainly because of their relatively large size and the similarity of their brains to the human brain. In fact, the differences between the anatomy of the posterior fossa of these brains are negligible under microscopic view regarding microsurgical techniques for cranial nerves around the brain stem [10, 14]. Moreover, these models are very satisfactory when familiarizing trainees with surgical techniques used in cranial approaches, especially around the CPA [15].

Despite all available data on animal models in otological and neurosurgical experiments and education, few publications can be found with an oto-neurosurgical background, especially the approach to the posterior fossa with cranial nerve exposure in the CPA and IAC [10, 13]. In humans, neurosurgical procedures for pathologies located at the CPA, such as meningioma or acoustic neuroma, always involve manipulations of the acoustic-facial bundle. These manipulations can carry a high risk of injury with functional loss postoperatively.

In our experiment, we used the pig as a nonliving model to demonstrate the feasibility of our approach to access cranial nerves, specifically, the facial and vestibulocochlear nerves in the CPA and IAC. The availability and low cost of the pig, combined with the ease of preparation made it a strong candidate. Also, the fact that we can reproduce this model in vivo later at our facility made the pig more convenient than other models such as sheep or cows. It is easy to obtain and maintain living pigs and to anesthetize them.

After a first look at the temporal bone of pigs, one may note that it has a completely different appearance from human temporal bone. The position and length of the EAC are markedly different in pigs, it is very long and narrow. In addition, the mastoid is difficult to identify in pigs, as it is poorly pneumatized and partially hidden beneath the atlanto-occipital joint. However, we used the pig’s cadavers as a model and after a closer look at the middle ear structures and tympanic membrane, we can observe that they are morphologically similar to the structures found in human ears, as has been described by several authors [4–6].

Skin incision and soft tissue dissection were performed in a posterior and inferior direction to the EAC, much as in humans, although thicker skin and massive muscle mass could be observed in the occipital and temporal area. This could be a technical disadvantage in the living model.

Regarding the anatomy of the skull base, it is highly similar to that of humans especially when it comes to the anatomy of cranial nerves and vessels in the posterior fossa [10–12]. Yet, drilling of that part of temporal bone covering the posterior fossa is difficult because the classic landmarks that are used in humans are either missing or have a different orientation. These include the linea temporalis (temporal line), EAC and mastoid cells which are used to define the correct location for the surgical procedure. We used other simple and easily identifiable landmarks instead, starting with the posterior wall of the EAC, the posterior arcade and the dura of the posterior fossa.

Access to the CPA, drilling of the IAC, and exposure of the complete course of the acoustic-facial bundle (CN VII/VIII) were accomplished in all specimens. The facial and vestibulocochlear nerves could be successfully exposed from their emergence in the CPA until they entered the IAC. These surgical manipulations represent important steps in all surgery involving the posterior fossa and CPA.

The strong similarities of the middle ear and its structures between humans and pigs recommend it as a possible model for otological research and education. The pig can be a strong candidate for oto-neurosurgical training concerning the CPA and IAC. Furthermore, this approach offers satisfactory exposure of the acoustic-facial-cranial nerve bundle with relatively straightforward and easily identifiable landmarks. Great similarities under the microscope could be noticed between the acoustic facial bundle in human and pigs (Fig 6). Hence, it can be further developed and used as a useful biological living animal model in research work concerning investigation and electrophysiological evaluation of cranial nerve injury.

## Conclusions

The outer appearance of the temporal bone of pigs differs markedly from that of humans; however, using our approach, the pig can offer a modeled surgical environment that can be used as an outline for surgical training of otologists and neurosurgeons using nonliving and living models. It can also be used in further research work on facial and cochlear nerve testing and the study of their neurophysiological behavior.
